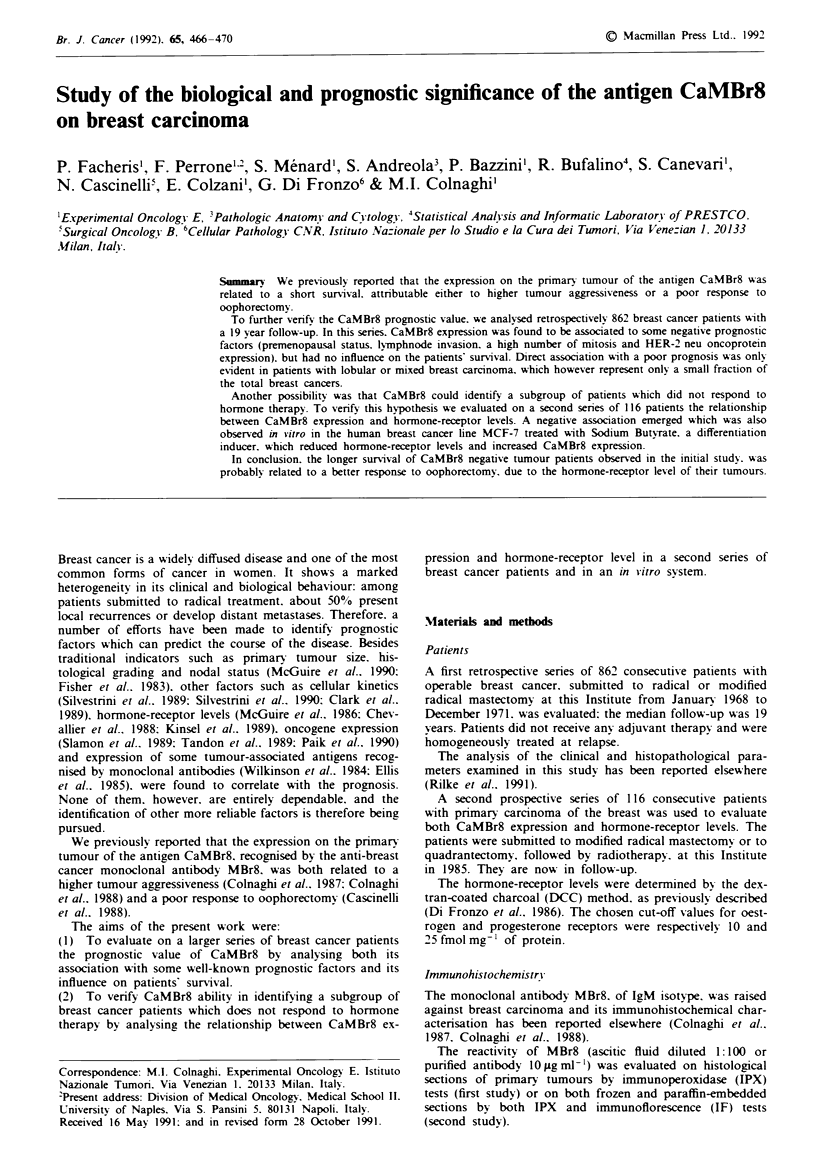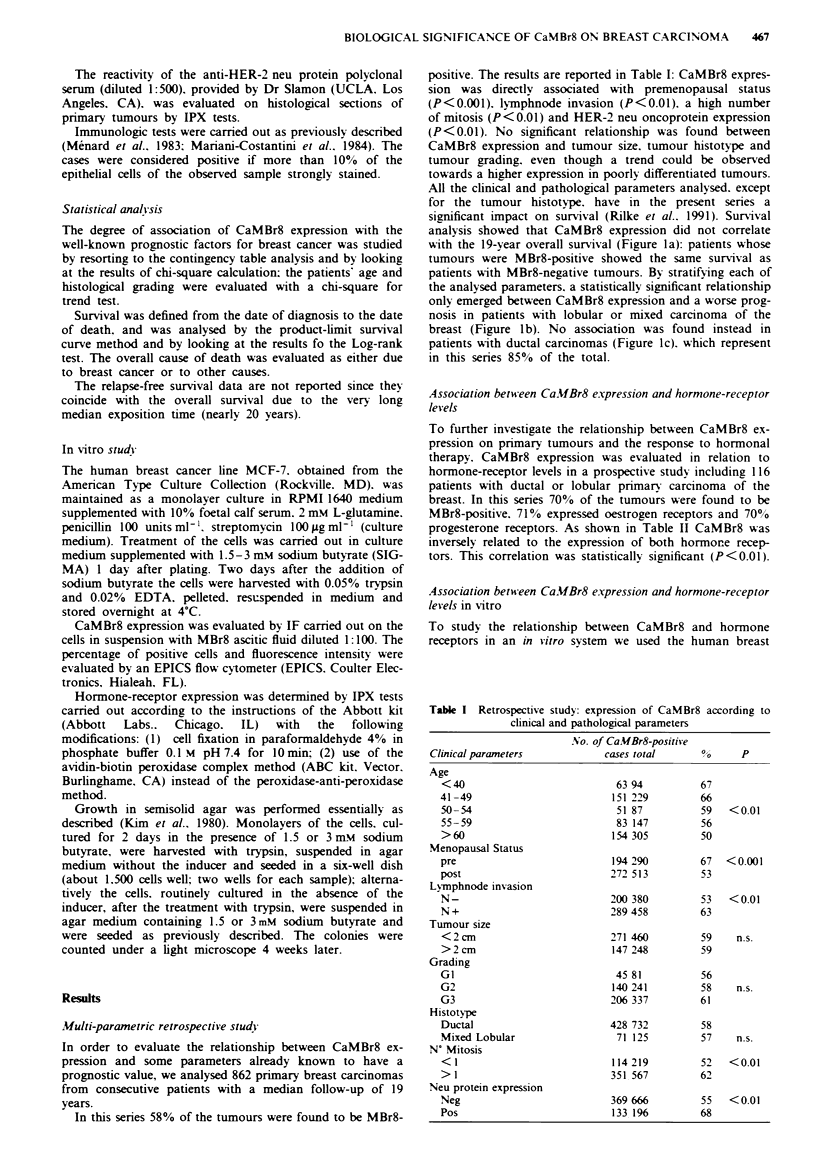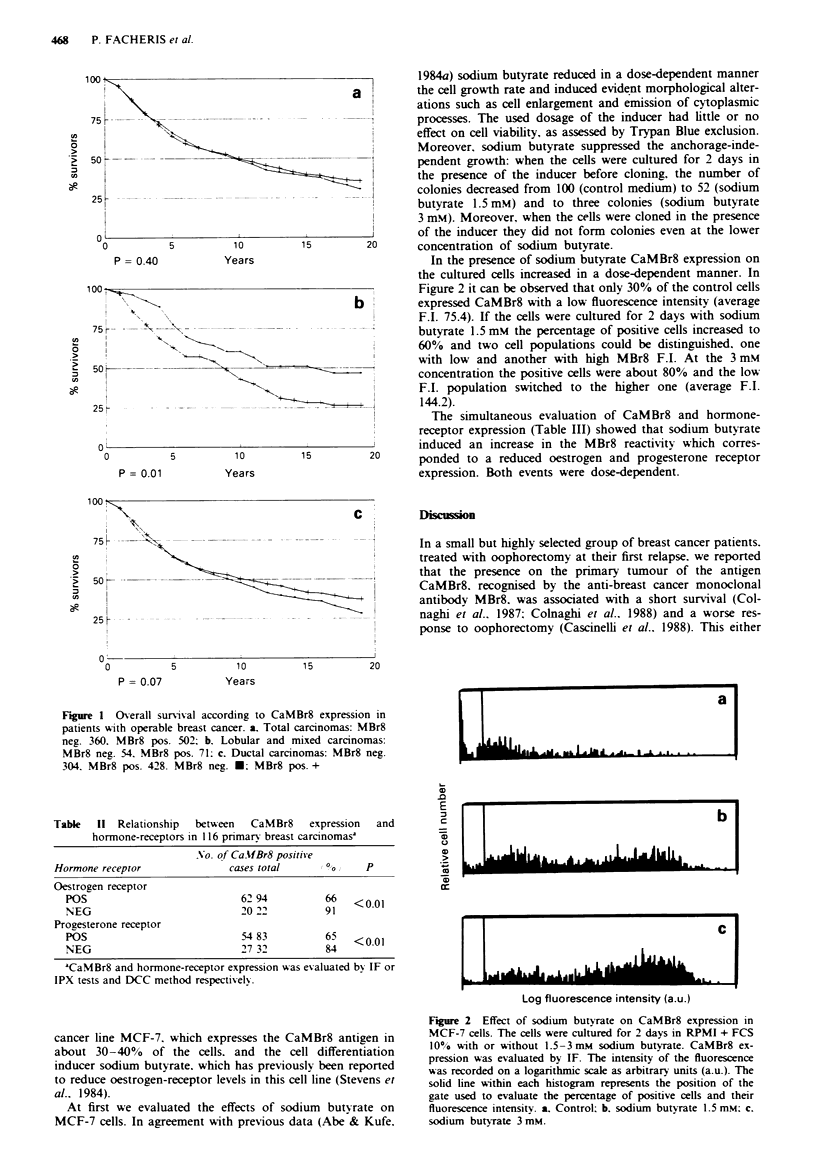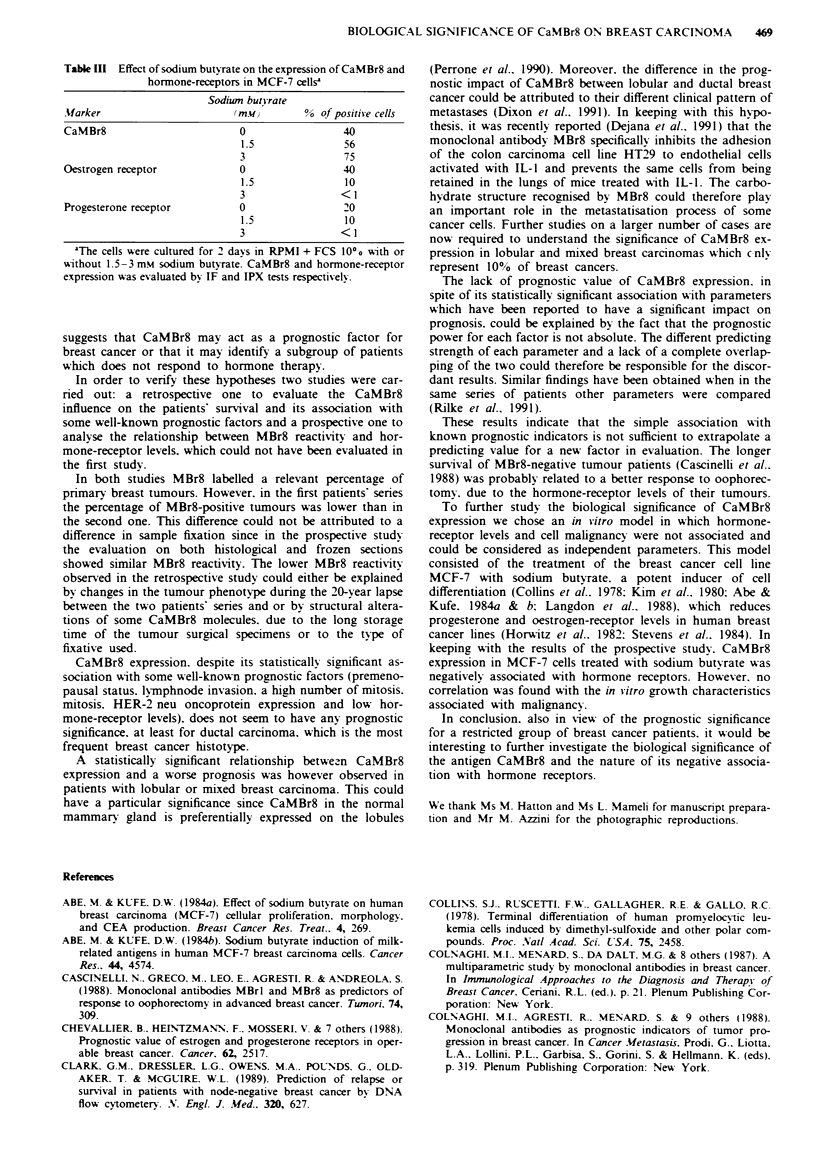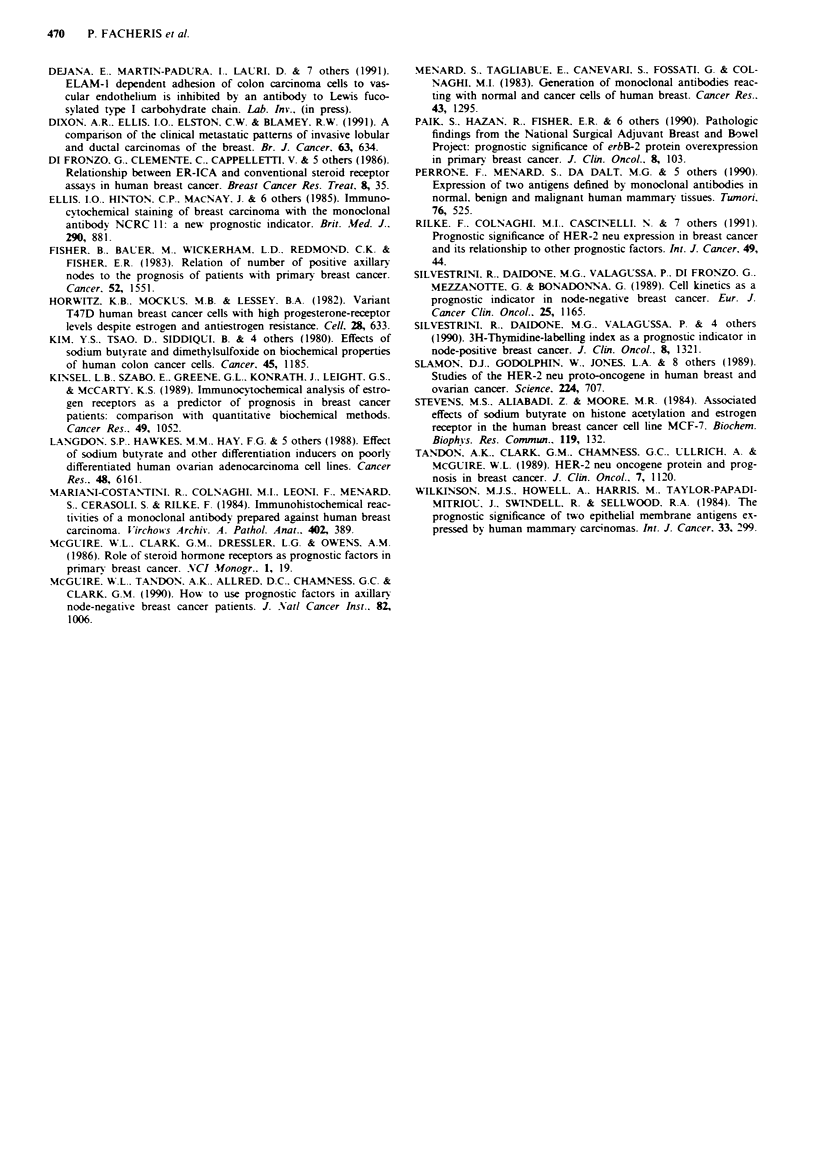# Study of the biological and prognostic significance of the antigen CaMBr8 on breast carcinoma.

**DOI:** 10.1038/bjc.1992.94

**Published:** 1992-03

**Authors:** P. Facheris, F. Perrone, S. Ménard, S. Andreola, P. Bazzini, R. Bufalino, S. Canevari, N. Cascinelli, E. Colzani, G. Di Fronzo

**Affiliations:** Experimental Oncology E, Istituto Nazionale per lo Studio e la Cura dei Tumori, Milan, Italy.

## Abstract

We previously reported that the expression on the primary tumour of the antigen CaMBr8 was related to a short survival, attributable either to higher tumour aggressiveness or a poor response to oophorectomy. To further verify the CaMBr8 prognostic value, we analysed retrospectively 862 breast cancer patients with a 19 year follow-up. In this series, CaMBr8 expression was found to be associated to some negative prognostic factors (premenopausal status, lymphnode invasion, a high number of mitosis and HER-2/neu oncoprotein expression), but had no influence on the patients' survival. Direct association with a poor prognosis was only evident in patients with lobular or mixed breast carcinoma, which however represent only a small fraction of the total breast cancers. Another possibility was that CaMBr8 could identify a subgroup of patients which did not respond to hormone therapy. To verify this hypothesis we evaluated on a second series of 116 patients the relationship between CaMBr8 expression and hormone-receptor levels. A negative association emerged which was also observed in vitro in the human breast cancer line MCF-7 treated with Sodium Butyrate, a differentiation inducer, which reduced hormone-receptor levels and increased CaMBr8 expression. In conclusion, the longer survival of CaMBr8 negative tumour patients observed in the initial study, was probably related to a better response to oophorectomy, due to the hormone-receptor level of their tumours.


Br. J. Cancer (1992), 65, 466-470 © Macmillan Press Ltd., 1992

### Study of the biological and prognostic significance of the antigen CaMBr8 on breast carcinoma

P. Facheris[1], F. Perrone[1,2], S. Ménard[1], S. Andreola[3], P. Bazzini[1], R. Bufalino[4], S. Canevari[1], N. Cascinelli[5], E. Colzani[1], G. Di Fronzo[6] & M.I. Colnaghi[1]

[1]*Experimental Oncology E,* [3]*Pathologic Anatomy and Cytology,* [4]*Statistical Analysis and Informatic Laboratory of PRESTCO,* [5]*Surgical Oncology B,* [6]*Cellular Pathology CNR, Istituto Nazionale per lo Studio e la Cura dei Tumori, Via Venezian 1, 20133 Milan, Italy.*

**Summary** We previously reported that the expression on the primary tumour of the antigen CaMBr8 was related to a short survival, attributable either to higher tumour aggressiveness or a poor response to oophorectomy.

To further verify the CaMBr8 prognostic value, we analysed retrospectively 862 breast cancer patients with a 19 year follow-up. In this series, CaMBr8 expression was found to be associated to some negative prognostic factors (premenopausal status, lymphnode invasion, a high number of mitosis and HER-2 neu oncoprotein expression), but had no influence on the patients' survival. Direct association with a poor prognosis was only evident in patients with lobular or mixed breast carcinoma, which however represent only a small fraction of the total breast cancers.

Another possibility was that CaMBr8 could identify a subgroup of patients which did not respond to hormone therapy. To verify this hypothesis we evaluated on a second series of 116 patients the relationship between CaMBr8 expression and hormone-receptor levels. A negative association emerged which was also observed *in vitro* in the human breast cancer line MCF-7 treated with Sodium Butyrate, a differentiation inducer, which reduced hormone-receptor levels and increased CaMBr8 expression.

In conclusion, the longer survival of CaMBr8 negative tumour patients observed in the initial study, was probably related to a better response to oophorectomy, due to the hormone-receptor level of their tumours.

Breast cancer is a widely diffused disease and one of the most common forms of cancer in women. It shows a marked heterogeneity in its clinical and biological behaviour: among patients submitted to radical treatment, about 50% present local recurrences or develop distant metastases. Therefore, a number of efforts have been made to identify prognostic factors which can predict the course of the disease. Besides traditional indicators such as primary tumour size, histological grading and nodal status (McGuire *et al.*, 1990; Fisher *et al.*, 1983), other factors such as cellular kinetics (Silvestrini *et al.*, 1989; Silvestrini *et al.*, 1990; Clark *et al.*, 1989), hormone-receptor levels (McGuire *et al.*, 1986; Chevallier *et al.*, 1988; Kinsel *et al.*, 1989), oncogene expression (Slamon *et al.*, 1989; Tandon *et al.*, 1989; Paik *et al.*, 1990) and expression of some tumour-associated antigens recognised by monoclonal antibodies (Wilkinson *et al.*, 1984; Ellis *et al.*, 1985), were found to correlate with the prognosis. None of them, however, are entirely dependable, and the identification of other more reliable factors is therefore being pursued.

We previously reported that the expression on the primary tumour of the antigen CaMBr8, recognised by the anti-breast cancer monoclonal antibody MBr8, was both related to a higher tumour aggressiveness (Colnaghi *et al.*, 1987; Colnaghi *et al.*, 1988) and a poor response to oophorectomy (Cascinelli *et al.*, 1988).

The aims of the present work were:

(1) To evaluate on a larger series of breast cancer patients the prognostic value of CaMBr8 by analysing both its association with some well-known prognostic factors and its influence on patients' survival.

(2) To verify CaMBr8 ability in identifying a subgroup of breast cancer patients which does not respond to hormone therapy by analysing the relationship between CaMBr8 expression and hormone-receptor level in a second series of breast cancer patients and in an *in vitro* system.

### Materials and methods

### Patients

A first retrospective series of 862 consecutive patients with operable breast cancer, submitted to radical or modified radical mastectomy at this Institute from January 1968 to December 1971, was evaluated; the median follow-up was 19 years. Patients did not receive any adjuvant therapy and were homogeneously treated at relapse.

The analysis of the clinical and histopathological parameters examined in this study has been reported elsewhere (Rilke *et al.*, 1991).

A second prospective series of 116 consecutive patients with primary carcinoma of the breast was used to evaluate both CaMBr8 expression and hormone-receptor levels. The patients were submitted to modified radical mastectomy or to quadrantectomy, followed by radiotherapy, at this Institute in 1985. They are now in follow-up.

The hormone-receptor levels were determined by the dextran-coated charcoal (DCC) method, as previously described (Di Fronzo *et al.*, 1986). The chosen cut-off values for oestrogen and progesterone receptors were respectively 10 and 25 $fmol\ mg^{-1}$ of protein.

### Immunohistochemistry

The monoclonal antibody MBr8, of IgM isotype, was raised against breast carcinoma and its immunohistochemical characterisation has been reported elsewhere (Colnaghi *et al.*, 1987, Colnaghi *et al.*, 1988).

The reactivity of MBr8 (ascitic fluid diluted 1:100 or purified antibody 10 $\mu g\ ml^{-1}$) was evaluated on histological sections of primary tumours by immunoperoxidase (IPX) tests (first study) or on both frozen and paraffin-embedded sections by both IPX and immunofluorescence (IF) tests (second study).

Correspondence: M.I. Colnaghi, Experimental Oncology E, Istituto Nazionale Tumori, Via Venezian 1, 20133 Milan, Italy.
[2]Present address: Division of Medical Oncology, Medical School II, University of Naples, Via S. Pansini 5, 80131 Napoli, Italy.
Received 16 May 1991; and in revised form 28 October 1991.

The reactivity of the anti-HER-2 neu protein polyclonal serum (diluted 1:500), provided by Dr Slamon (UCLA, Los Angeles, CA), was evaluated on histological sections of primary tumours by IPX tests.

Immunologic tests were carried out as previously described (Ménard *et al.*, 1983; Mariani-Costantini *et al.*, 1984). The cases were considered positive if more than 10% of the epithelial cells of the observed sample strongly stained.

*Statistical analysis*

The degree of association of CaMBr8 expression with the well-known prognostic factors for breast cancer was studied by resorting to the contingency table analysis and by looking at the results of chi-square calculation; the patients' age and histological grading were evaluated with a chi-square for trend test.

Survival was defined from the date of diagnosis to the date of death, and was analysed by the product-limit survival curve method and by looking at the results fo the Log-rank test. The overall cause of death was evaluated as either due to breast cancer or to other causes.

The relapse-free survival data are not reported since they coincide with the overall survival due to the very long median exposition time (nearly 20 years).

In vitro *study*

The human breast cancer line MCF-7, obtained from the American Type Culture Collection (Rockville, MD), was maintained as a monolayer culture in RPMI 1640 medium supplemented with 10% foetal calf serum, 2 mM L-glutamine, penicillin 100 units $ml^{-1}$, streptomycin 100 $\mu g\ ml^{-1}$ (culture medium). Treatment of the cells was carried out in culture medium supplemented with 1.5–3 mM sodium butyrate (SIGMA) 1 day after plating. Two days after the addition of sodium butyrate the cells were harvested with 0.05% trypsin and 0.02% EDTA, pelleted, resuspended in medium and stored overnight at 4°C.

CaMBr8 expression was evaluated by IF carried out on the cells in suspension with MBr8 ascitic fluid diluted 1:100. The percentage of positive cells and fluorescence intensity were evaluated by an EPICS flow cytometer (EPICS, Coulter Electronics, Hialeah, FL).

Hormone-receptor expression was determined by IPX tests carried out according to the instructions of the Abbott kit (Abbott Labs., Chicago, IL) with the following modifications: (1) cell fixation in paraformaldehyde 4% in phosphate buffer 0.1 M pH 7.4 for 10 min; (2) use of the avidin-biotin peroxidase complex method (ABC kit, Vector, Burlinghame, CA) instead of the peroxidase-anti-peroxidase method.

Growth in semisolid agar was performed essentially as described (Kim *et al.*, 1980). Monolayers of the cells, cultured for 2 days in the presence of 1.5 or 3 mM sodium butyrate, were harvested with trypsin, suspended in agar medium without the inducer and seeded in a six-well dish (about 1,500 cells well; two wells for each sample); alternatively the cells, routinely cultured in the absence of the inducer, after the treatment with trypsin, were suspended in agar medium containing 1.5 or 3 mM sodium butyrate and were seeded as previously described. The colonies were counted under a light microscope 4 weeks later.

### Results

*Multi-parametric retrospective study*

In order to evaluate the relationship between CaMBr8 expression and some parameters already known to have a prognostic value, we analysed 862 primary breast carcinomas from consecutive patients with a median follow-up of 19 years.

In this series 58% of the tumours were found to be MBr8-positive. The results are reported in Table I: CaMBr8 expression was directly associated with premenopausal status ($P<0.001$), lymphnode invasion ($P<0.01$), a high number of mitosis ($P<0.01$) and HER-2 neu oncoprotein expression ($P<0.01$). No significant relationship was found between CaMBr8 expression and tumour size, tumour histotype and tumour grading, even though a trend could be observed towards a higher expression in poorly differentiated tumours. All the clinical and pathological parameters analysed, except for the tumour histotype, have in the present series a significant impact on survival (Rilke *et al.*, 1991). Survival analysis showed that CaMBr8 expression did not correlate with the 19-year overall survival (Figure 1a): patients whose tumours were MBr8-positive showed the same survival as patients with MBr8-negative tumours. By stratifying each of the analysed parameters, a statistically significant relationship only emerged between CaMBr8 expression and a worse prognosis in patients with lobular or mixed carcinoma of the breast (Figure 1b). No association was found instead in patients with ductal carcinomas (Figure 1c), which represent in this series 85% of the total.

*Association between CaMBr8 expression and hormone-receptor levels*

To further investigate the relationship between CaMBr8 expression on primary tumours and the response to hormonal therapy, CaMBr8 expression was evaluated in relation to hormone-receptor levels in a prospective study including 116 patients with ductal or lobular primary carcinoma of the breast. In this series 70% of the tumours were found to be MBr8-positive, 71% expressed oestrogen receptors and 70% progesterone receptors. As shown in Table II CaMBr8 was inversely related to the expression of both hormone receptors. This correlation was statistically significant ($P<0.01$).

*Association between CaMBr8 expression and hormone-receptor levels* in vitro

To study the relationship between CaMBr8 and hormone receptors in an *in vitro* system we used the human breast

**Table I** Retrospective study: expression of CaMBr8 according to clinical and pathological parameters

| *Clinical parameters* | *No. of CaMBr8-positive cases total* | % | *P* |
|---|---|---|---|
| Age | | | |
| <40 | 63 94 | 67 | |
| 41–49 | 151 229 | 66 | |
| 50–54 | 51 87 | 59 | <0.01 |
| 55–59 | 83 147 | 56 | |
| >60 | 154 305 | 50 | |
| Menopausal Status | | | |
| pre | 194 290 | 67 | <0.001 |
| post | 272 513 | 53 | |
| Lymphnode invasion | | | |
| N− | 200 380 | 53 | <0.01 |
| N+ | 289 458 | 63 | |
| Tumour size | | | |
| <2 cm | 271 460 | 59 | n.s. |
| >2 cm | 147 248 | 59 | |
| Grading | | | |
| G1 | 45 81 | 56 | |
| G2 | 140 241 | 58 | n.s. |
| G3 | 206 337 | 61 | |
| Histotype | | | |
| Ductal | 428 732 | 58 | |
| Mixed Lobular | 71 125 | 57 | n.s. |
| N° Mitosis | | | |
| <1 | 114 219 | 52 | <0.01 |
| >1 | 351 567 | 62 | |
| Neu protein expression | | | |
| Neg | 369 666 | 55 | <0.01 |
| Pos | 133 196 | 68 | |

**Figure 1** Overall survival according to CaMBr8 expression in patients with operable breast cancer. **a**, Total carcinomas: MBr8 neg. 360, MBr8 pos. 502; **b**, Lobular and mixed carcinomas: MBr8 neg. 54, MBr8 pos. 71; **c**, Ductal carcinomas: MBr8 neg. 304, MBr8 pos. 428. MBr8 neg. ■; MBr8 pos. +

**Table II** Relationship between CaMBr8 expression and hormone-receptors in 116 primary breast carcinomas[a]

| Hormone receptor | No. of CaMBr8 positive cases total | (%) | P |
|---|---|---|---|
| Oestrogen receptor | | | |
| POS | 62 94 | 66 | <0.01 |
| NEG | 20 22 | 91 | |
| Progesterone receptor | | | |
| POS | 54 83 | 65 | <0.01 |
| NEG | 27 32 | 84 | |

[a]CaMBr8 and hormone-receptor expression was evaluated by IF or IPX tests and DCC method respectively.

cancer line MCF-7, which expresses the CaMBr8 antigen in about 30–40% of the cells, and the cell differentiation inducer sodium butyrate, which has previously been reported to reduce oestrogen-receptor levels in this cell line (Stevens *et al.*, 1984).

At first we evaluated the effects of sodium butyrate on MCF-7 cells. In agreement with previous data (Abe & Kufe, 1984*a*) sodium butyrate reduced in a dose-dependent manner the cell growth rate and induced evident morphological alterations such as cell enlargement and emission of cytoplasmic processes. The used dosage of the inducer had little or no effect on cell viability, as assessed by Trypan Blue exclusion. Moreover, sodium butyrate suppressed the anchorage-independent growth: when the cells were cultured for 2 days in the presence of the inducer before cloning, the number of colonies decreased from 100 (control medium) to 52 (sodium butyrate 1.5 mM) and to three colonies (sodium butyrate 3 mM). Moreover, when the cells were cloned in the presence of the inducer they did not form colonies even at the lower concentration of sodium butyrate.

In the presence of sodium butyrate CaMBr8 expression on the cultured cells increased in a dose-dependent manner. In Figure 2 it can be observed that only 30% of the control cells expressed CaMBr8 with a low fluorescence intensity (average F.I. 75.4). If the cells were cultured for 2 days with sodium butyrate 1.5 mM the percentage of positive cells increased to 60% and two cell populations could be distinguished, one with low and another with high MBr8 F.I. At the 3 mM concentration the positive cells were about 80% and the low F.I. population switched to the higher one (average F.I. 144.2).

The simultaneous evaluation of CaMBr8 and hormone-receptor expression (Table III) showed that sodium butyrate induced an increase in the MBr8 reactivity which corresponded to a reduced oestrogen and progesterone receptor expression. Both events were dose-dependent.

### Discussion

In a small but highly selected group of breast cancer patients, treated with oophorectomy at their first relapse, we reported that the presence on the primary tumour of the antigen CaMBr8, recognised by the anti-breast cancer monoclonal antibody MBr8, was associated with a short survival (Colnaghi *et al.*, 1987; Colnaghi *et al.*, 1988) and a worse response to oophorectomy (Cascinelli *et al.*, 1988). This either

**Figure 2** Effect of sodium butyrate on CaMBr8 expression in MCF-7 cells. The cells were cultured for 2 days in RPMI + FCS 10% with or without 1.5–3 mM sodium butyrate. CaMBr8 expression was evaluated by IF. The intensity of the fluorescence was recorded on a logarithmic scale as arbitrary units (a.u.). The solid line within each histogram represents the position of the gate used to evaluate the percentage of positive cells and their fluorescence intensity. **a**, Control; **b**, sodium butyrate 1.5 mM; **c**, sodium butyrate 3 mM.

**Table III** Effect of sodium butyrate on the expression of CaMBr8 and hormone-receptors in MCF-7 cells[a]

| *Marker* | *Sodium butyrate (mM)* | *% of positive cells* |
|---|---|---|
| CaMBr8 | 0 | 40 |
| | 1.5 | 56 |
| | 3 | 75 |
| Oestrogen receptor | 0 | 40 |
| | 1.5 | 10 |
| | 3 | <1 |
| Progesterone receptor | 0 | 20 |
| | 1.5 | 10 |
| | 3 | <1 |

[a]The cells were cultured for 2 days in RPMI + FCS 10% with or without 1.5–3 mM sodium butyrate. CaMBr8 and hormone-receptor expression was evaluated by IF and IPX tests respectively.

suggests that CaMBr8 may act as a prognostic factor for breast cancer or that it may identify a subgroup of patients which does not respond to hormone therapy.

In order to verify these hypotheses two studies were carried out: a retrospective one to evaluate the CaMBr8 influence on the patients' survival and its association with some well-known prognostic factors and a prospective one to analyse the relationship between MBr8 reactivity and hormone-receptor levels, which could not have been evaluated in the first study.

In both studies MBr8 labelled a relevant percentage of primary breast tumours. However, in the first patients' series the percentage of MBr8-positive tumours was lower than in the second one. This difference could not be attributed to a difference in sample fixation since in the prospective study the evaluation on both histological and frozen sections showed similar MBr8 reactivity. The lower MBr8 reactivity observed in the retrospective study could either be explained by changes in the tumour phenotype during the 20-year lapse between the two patients' series and or by structural alterations of some CaMBr8 molecules, due to the long storage time of the tumour surgical specimens or to the type of fixative used.

CaMBr8 expression, despite its statistically significant association with some well-known prognostic factors (premenopausal status, lymphnode invasion, a high number of mitosis, mitosis, HER-2 neu oncoprotein expression and low hormone-receptor levels), does not seem to have any prognostic significance, at least for ductal carcinoma, which is the most frequent breast cancer histotype.

A statistically significant relationship between CaMBr8 expression and a worse prognosis was however observed in patients with lobular or mixed breast carcinoma. This could have a particular significance since CaMBr8 in the normal mammary gland is preferentially expressed on the lobules (Perrone *et al.*, 1990). Moreover, the difference in the prognostic impact of CaMBr8 between lobular and ductal breast cancer could be attributed to their different clinical pattern of metastases (Dixon *et al.*, 1991). In keeping with this hypothesis, it was recently reported (Dejana *et al.*, 1991) that the monoclonal antibody MBr8 specifically inhibits the adhesion of the colon carcinoma cell line HT29 to endothelial cells activated with IL-1 and prevents the same cells from being retained in the lungs of mice treated with IL-1. The carbohydrate structure recognised by MBr8 could therefore play an important role in the metastatisation process of some cancer cells. Further studies on a larger number of cases are now required to understand the significance of CaMBr8 expression in lobular and mixed breast carcinomas which only represent 10% of breast cancers.

The lack of prognostic value of CaMBr8 expression, in spite of its statistically significant association with parameters which have been reported to have a significant impact on prognosis, could be explained by the fact that the prognostic power for each factor is not absolute. The different predicting strength of each parameter and a lack of a complete overlapping of the two could therefore be responsible for the discordant results. Similar findings have been obtained when in the same series of patients other parameters were compared (Rilke *et al.*, 1991).

These results indicate that the simple association with known prognostic indicators is not sufficient to extrapolate a predicting value for a new factor in evaluation. The longer survival of MBr8-negative tumour patients (Cascinelli *et al.*, 1988) was probably related to a better response to oophorectomy, due to the hormone-receptor levels of their tumours.

To further study the biological significance of CaMBr8 expression we chose an *in vitro* model in which hormone-receptor levels and cell malignancy were not associated and could be considered as independent parameters. This model consisted of the treatment of the breast cancer cell line MCF-7 with sodium butyrate, a potent inducer of cell differentiation (Collins *et al.*, 1978; Kim *et al.*, 1980; Abe & Kufe, 1984*a* & *b*; Langdon *et al.*, 1988), which reduces progesterone and oestrogen-receptor levels in human breast cancer lines (Horwitz *et al.*, 1982; Stevens *et al.*, 1984). In keeping with the results of the prospective study, CaMBr8 expression in MCF-7 cells treated with sodium butyrate was negatively associated with hormone receptors. However, no correlation was found with the *in vitro* growth characteristics associated with malignancy.

In conclusion, also in view of the prognostic significance for a restricted group of breast cancer patients, it would be interesting to further investigate the biological significance of the antigen CaMBr8 and the nature of its negative association with hormone receptors.

We thank Ms M. Hatton and Ms L. Mameli for manuscript preparation and Mr M. Azzini for the photographic reproductions.

### References

ABE, M. & KUFE, D.W. (1984*a*). Effect of sodium butyrate on human breast carcinoma (MCF-7) cellular proliferation, morphology, and CEA production. *Breast Cancer Res. Treat.*, **4**, 269.

ABE, M. & KUFE, D.W. (1984*b*). Sodium butyrate induction of milk-related antigens in human MCF-7 breast carcinoma cells. *Cancer Res.*, **44**, 4574.

CASCINELLI, N., GRECO, M., LEO, E., AGRESTI, R. & ANDREOLA, S. (1988). Monoclonal antibodies MBr1 and MBr8 as predictors of response to oophorectomy in advanced breast cancer. *Tumori*, **74**, 309.

CHEVALLIER, B., HEINTZMANN, F., MOSSERI, V. & 7 others (1988). Prognostic value of estrogen and progesterone receptors in operable breast cancer. *Cancer*, **62**, 2517.

CLARK, G.M., DRESSLER, L.G., OWENS, M.A., POUNDS, G., OLDAKER, T. & MCGUIRE, W.L. (1989). Prediction of relapse or survival in patients with node-negative breast cancer by DNA flow cytometery. *N. Engl. J. Med.*, **320**, 627.

COLLINS, S.J., RUSCETTI, F.W., GALLAGHER, R.E. & GALLO, R.C. (1978). Terminal differentiation of human promyelocytic leukemia cells induced by dimethyl-sulfoxide and other polar compounds. *Proc. Natl Acad. Sci. USA*, **75**, 2458.

COLNAGHI, M.I., MENARD, S., DA DALT, M.G. & 8 others (1987). A multiparametric study by monoclonal antibodies in breast cancer. In *Immunological Approaches to the Diagnosis and Therapy of Breast Cancer*, Ceriani, R.L. (ed.), p. 21. Plenum Publishing Corporation: New York.

COLNAGHI, M.I., AGRESTI, R., MENARD, S. & 9 others (1988). Monoclonal antibodies as prognostic indicators of tumor progression in breast cancer. In *Cancer Metastasis*, Prodi, G., Liotta, L.A., Lollini, P.L., Garbisa, S., Gorini, S. & Hellmann, K. (eds), p. 319. Plenum Publishing Corporation: New York.

DEJANA, E., MARTIN-PADURA, I., LAURI, D. & 7 others (1991). ELAM-1 dependent adhesion of colon carcinoma cells to vascular endothelium is inhibited by an antibody to Lewis fucosylated type I carbohydrate chain. *Lab. Inv.*, (in press).

DIXON, A.R., ELLIS, I.O., ELSTON, C.W. & BLAMEY, R.W. (1991). A comparison of the clinical metastatic patterns of invasive lobular and ductal carcinomas of the breast. *Br. J. Cancer*, **63**, 634.

DI FRONZO, G., CLEMENTE, C., CAPPELLETTI, V. & 5 others (1986). Relationship between ER-ICA and conventional steroid receptor assays in human breast cancer. *Breast Cancer Res. Treat.* **8**, 35.

ELLIS, I.O., HINTON, C.P., MACNAY, J. & 6 others (1985). Immunocytochemical staining of breast carcinoma with the monoclonal antibody NCRC 11: a new prognostic indicator. *Brit. Med. J.*, **290**, 881.

FISHER, B., BAUER, M., WICKERHAM, L.D., REDMOND, C.K. & FISHER, E.R. (1983). Relation of number of positive axillary nodes to the prognosis of patients with primary breast cancer. *Cancer*, **52**, 1551.

HORWITZ, K.B., MOCKUS, M.B. & LESSEY, B.A. (1982). Variant T47D human breast cancer cells with high progesterone-receptor levels despite estrogen and antiestrogen resistance. *Cell*, **28**, 633.

KIM, Y.S., TSAO, D., SIDDIQUI, B. & 4 others (1980). Effects of sodium butyrate and dimethylsulfoxide on biochemical properties of human colon cancer cells. *Cancer*, **45**, 1185.

KINSEL, L.B., SZABO, E., GREENE, G.L., KONRATH, J., LEIGHT, G.S., & McCARTY, K.S. (1989). Immunocytochemical analysis of estrogen receptors as a predictor of prognosis in breast cancer patients: comparison with quantitative biochemical methods. *Cancer Res.*, **49**, 1052.

LANGDON, S.P., HAWKES, M.M., HAY, F.G. & 5 others (1988). Effect of sodium butyrate and other differentiation inducers on poorly differentiated human ovarian adenocarcinoma cell lines. *Cancer Res.*, **48**, 6161.

MARIANI-COSTANTINI, R., COLNAGHI, M.I., LEONI, F., MENARD, S., CERASOLI, S. & RILKE, F. (1984). Immunohistochemical reactivities of a monoclonal antibody prepared against human breast carcinoma. *Virchows Archiv. A. Pathol. Anat.*, **402**, 389.

McGUIRE, W.L., CLARK, G.M., DRESSLER, L.G. & OWENS, A.M. (1986). Role of steroid hormone receptors as prognostic factors in primary breast cancer. *NCI Monogr.*, **1**, 19.

McGUIRE, W.L., TANDON, A.K., ALLRED, D.C., CHAMNESS, G.C. & CLARK, G.M. (1990). How to use prognostic factors in axillary node-negative breast cancer patients. *J. Natl Cancer Inst.*, **82**, 1006.

MENARD, S., TAGLIABUE, E., CANEVARI, S., FOSSATI, G. & COLNAGHI, M.I. (1983). Generation of monoclonal antibodies reacting with normal and cancer cells of human breast. *Cancer Res.*, **43**, 1295.

PAIK, S., HAZAN, R., FISHER, E.R. & 6 others (1990). Pathologic findings from the National Surgical Adjuvant Breast and Bowel Project: prognostic significance of *erb*B-2 protein overexpression in primary breast cancer. *J. Clin. Oncol.*, **8**, 103.

PERRONE, F., MENARD, S., DA DALT, M.G. & 5 others (1990). Expression of two antigens defined by monoclonal antibodies in normal, benign and malignant human mammary tissues. *Tumori*, **76**, 525.

RILKE, F., COLNAGHI, M.I., CASCINELLI, N. & 7 others (1991). Prognostic significance of HER-2 neu expression in breast cancer and its relationship to other prognostic factors. *Int. J. Cancer*, **49**, 44.

SILVESTRINI, R., DAIDONE, M.G., VALAGUSSA, P., DI FRONZO, G., MEZZANOTTE, G. & BONADONNA, G. (1989). Cell kinetics as a prognostic indicator in node-negative breast cancer. *Eur. J. Cancer Clin. Oncol.*, **25**, 1165.

SILVESTRINI, R., DAIDONE, M.G., VALAGUSSA, P. & 4 others (1990). 3H-Thymidine-labelling index as a prognostic indicator in node-positive breast cancer. *J. Clin. Oncol.*, **8**, 1321.

SLAMON, D.J., GODOLPHIN, W., JONES, L.A. & 8 others (1989). Studies of the HER-2 neu proto-oncogene in human breast and ovarian cancer. *Science*, **224**, 707.

STEVENS, M.S., ALIABADI, Z. & MOORE, M.R. (1984). Associated effects of sodium butyrate on histone acetylation and estrogen receptor in the human breast cancer cell line MCF-7. *Biochem. Biophys. Res. Commun.*, **119**, 132.

TANDON, A.K., CLARK, G.M., CHAMNESS, G.C., ULLRICH, A. & McGUIRE, W.L. (1989). HER-2 neu oncogene protein and prognosis in breast cancer. *J. Clin. Oncol.*, **7**, 1120.

WILKINSON, M.J.S., HOWELL, A., HARRIS, M., TAYLOR-PAPADIMITRIOU, J., SWINDELL, R. & SELLWOOD, R.A. (1984). The prognostic significance of two epithelial membrane antigens expressed by human mammary carcinomas. *Int. J. Cancer*, **33**, 299.